# Stage at which riluzole treatment prolongs survival in patients with amyotrophic lateral sclerosis: a retrospective analysis of data from a dose-ranging study

**DOI:** 10.1016/S1474-4422(18)30054-1

**Published:** 2018-05

**Authors:** Ton Fang, Ahmad Al Khleifat, Jacques-Henri Meurgey, Ashley Jones, P Nigel Leigh, Gilbert Bensimon, Ammar Al-Chalabi

**Affiliations:** a King's College London, Maurice Wohl Clinical Neuroscience Institute, Department of Basic and Clinical Neuroscience, Institute of Psychiatry, Psychology & Neuroscience, London, UK; b King's College Hospital, Denmark Hill, London, UK; c Department of Neurology, Brighton and Sussex Medical School, Sussex, UK; d Department of Pharmacology, Hôpital Pitié-Salpêtrière, Paris, France

## Abstract

**Background:**

Riluzole is the only drug to prolong survival for amyotrophic lateral sclerosis (ALS) and, at a dose of 100 mg, was associated with a 35% reduction in mortality in a clinical trial. A key question is whether the survival benefit occurs at an early stage of disease, late stage, or is spread throughout the course of the disease. To address this question, we used the King's clinical staging system to do a retrospective analysis of data from the original dose-ranging clinical trial of riluzole.

**Methods:**

In the original dose-ranging trial, patients were enrolled between December, 1992, and November, 1993, in Belgium, France, Germany, Spain, Canada, the USA, and the UK if they had probable or definite ALS as defined by the El Escorial criteria. The censor date for the riluzole survival data was set as the original study end date of Dec 31, 1994. For this analysis, King's clinical ALS stage was estimated from the electronic case record data of the modified Norris scale, UK Medical Research Council score for muscle strength, El Escorial category, vital capacity, and gastrostomy insertion data. The lowest allocated stage was 2 because the original trial only included patients with probable or definite ALS. We used a χ^2^ test to assess the independence of stage at trial enrolment and treatment group, Kaplan-Meier product limit distribution to test the transition from each stage to subsequent stages, and Cox regression to confirm an effect of treatment group on time in stage, controlling for covariates. We did sensitivity analyses by combining treatment groups, using alternative strategies to stage, stratifying by stage at trial enrolment, and using multistate outcome analysis of treatments (MOAT).

**Findings:**

We analysed the case records of all 959 participants from the original dose-ranging trial, 237 assigned to 50 mg/day riluzole, 236 to 100 mg/day, 244 to 200 mg/day, and 242 to daily placebo. Clinical stage at enrolment did not significantly differ between treatment groups (p=0·22). Time in stage 4 was longer for patients receiving 100 mg/day riluzole than for those receiving placebo (hazard ratio [HR] 0·55, 95% CI 0·36–0·83; log-rank p=0·037). Combining treatment groups and stratifying by stage at enrolment showed a similar result (HR 0·638, 95% CI 0·464–0·878; p=0·006), as did analysis with MOAT where the mean number of days spent in stage 4 was numerically higher for patients given riluzole at higher doses compared with patients receiving placebo. Time from stages 2 or 3 to subsequent stages or death did not differ between riluzole treatment groups and placebo (p=0·83 for stage 2 and 0·88 for stage 3).

**Interpretation:**

We showed that riluzole prolongs survival in the last clinical stage of ALS; this finding needs to be confirmed in a prospective study, and treatment effects at stage 1 still need to be analysed. The ALS stage at which benefit occurs is important for counselling of patients before starting treatment. Staging should be used in future ALS clinical trials to assess the stage at which survival benefit occurs, and a similar approach could be used for other neurodegenerative diseases.

**Funding:**

NIHR Maudsley Biomedical Research Centre, The European Union Joint Programme on Neurodegeneration, and the King's Summer Undergraduate Studentship.

## Introduction

Amyotrophic lateral sclerosis (ALS) is a fatal neurodegenerative disorder of upper and lower motor neurons that causes progressive paralysis and eventually death from respiratory failure.^1^ The course of ALS varies substantially between people, so that although median survival from symptom onset is only 27·5 months for those with bulbar onset (IQR 19·8–39·5) and 35·9 months (22·9–56·4) for those with spinal onset,^2^ the range of survival length is wide, sometimes more than 20 years. This variability complicates the analysis of clinical trials, since time is used as a proxy for disease progression, but the wide variation means that in some people there will have been little change, which potentially reduces the power to detect an effect of treatment. To mitigate this variability, most ALS trials have strict entry criteria that aim to exclude the most slowly progressing patients. Disease staging is another approach, because it provides a framework for measuring disease progression regardless of whether the disease is aggressive or slow in any one individual. Several methods of staging ALS have been proposed, but the most widely used systems are the Milano-Torino functional staging system^3^ and the King's clinical staging system,^4^ both of which can be derived from standard clinical observations.

**Research in context**

**Evidence before this study**

It is important to know whether the survival benefit of riluzole in patients with amyotrophic lateral sclerosis (ALS) occurs early, late, or throughout the course of the disease to enable proper counselling of patients. We searched PubMed for reports published at any date up to July 31, 2017, using the terms “riluzole”; “amyotrophic lateral sclerosis”, “motor neuron disease”, “motor neurone disease”, “ALS”, or “MND”; and “stage” or “staging”. We included randomised, placebo-controlled trials in patients with ALS that involved riluzole alone and studies of clinical staging in ALS. We identified two trials, both of which were randomised, placebo-controlled studies and one of which included sufficient data for retrospective staging. We approached the commercial owners of the riluzole clinical trial data for full academic access to the trial database.

**Added value of this study**

By use of the King's clinical ALS staging system, we showed in a retrospective analsysis that riluzole prolonged stage 4 ALS in a dose-dependent manner, with no apparent prolongation of stages 2 or 3. We were unable to determine if there was an effect on stage 1.

**Implications of all the available evidence**

The timing of any benefit from riluzole affects the information that needs to be given to patients, because they are likely to interpret the benefit of prolongation of a later stage of disease as different from the benefit of prolonging an early stage, or prolongation of the disease course in general. The timing of benefit also has implications for health economics because the later stages of ALS are associated with higher costs than earlier stages, and therefore prolonging stage 4 is more costly than prolonging stages 1 or 2. Further studies are needed to determine if there is a survival benefit of riluzole in stage 1 ALS, and staging analyses should be used in future clinical trials of treatments in patients with ALS and other neurodegenerative diseases.

King's clinical stages range from 1 (early disease) to 4 (late disease), with stage 5 being death. The state of the patient's motor system is assessed using the El Escorial criteria domains of bulbar, upper limb, and lower limb.^5^ Stages 1, 2, and 3 correspond to involvement of one, two, or three domains respectively, as evidenced by symptoms or examination findings. Stage 4 corresponds to nutritional failure (10% of premorbid weight loss because of dysphagia), or substantial respiratory failure (fulfilling guidelines for needing non-invasive ventilation). King's stage can be estimated from the revised ALS functional rating scale (ALSFRS-R) with a high correspondence to actual clinical stage, making it useful for retrospective analyses.^6^, ^7^ King's clinical staging system has been validated by confirmation in several populations and by correlation with biomarkers, and has been used to assess the timing of cognitive changes in ALS and for health-economics analyses.^8^

Riluzole, a glutamatergic antagonist, is the only disease-modifying treatment shown to extend life in patients with ALS, associated with a 38·6% reduction in mortality in an efficacy trial^9^ and a 35% improvement in survival with the 100 mg dose in a dose-ranging trial.^10^ Riluzole has not shown an apparent effect on function, but any functional effect is difficult to assess in an ALS clinical trial because patients with the worst function tend to drop out of the study,^11^ either because travel becomes too onerous, or through death. As a result, only patients with relatively preserved function remain, which reduces the statistical power to detect an effect between treatment groups. A key question therefore is whether riluzole extends life throughout the course of ALS, or only at an early or late stage of the disease. From a patient's perspective, there is a major difference between a drug that prolongs the early disease stages and a drug that prolongs later disease stages. From a health-economics standpoint, a survival benefit from extending early stages of disease might be preferable to prolongation at later stages where management of symptoms can be more expensive.^7^ To understand the relationship between the survival benefit of riluzole and disease stage, we retrospectively did a clinical staging analysis of results from the original dose-ranging study of riluzole.^10^

## Methods

### Participants and data collection

Patients in Belgium, France, Germany, Spain, Canada, the USA, and the UK with probable or definite ALS as defined by the El Escorial criteria were eligible for the original riluzole dose-ranging study, and enrolled between December, 1992, and November, 1993.^10^ In this retrospective analysis, we extracted data from the electronic case record forms of that study. The censor date for the riluzole survival data was set as the original study end date, Dec 31, 1994. To exclude an artifactual explanation for the findings, patient case record data from a trial of lithium carbonate in ALS (LiCALS; ISRCTN 83178718) were also tested for comparison.^12^

In this analysis, we staged participants retrospectively from the clinical trial data collected at each study visit using an algorithm. For the LiCALS study, an algorithm based on the ALSFRS-R had a correlation of 92% with actual clinical stage.^6^ However, the riluzole dose-ranging study data were collected before the ALSFRS-R had been developed, and we therefore used the same principles to develop a corresponding algorithm based on functional scores from electronic case-records to assign King's clinical staging. Affected domains were established following specific criteria (appendix), using questions on the modified Norris scales, UK Medical Research Council muscle strength scores, El Escorial category, gastrostomy data, and vital capacity. Tracheostomy and intubation were classified as equivalent to death for the purposes of analysis. Allocation to stage 4 requires either nutritional failure sufficient to require gastrostomy, or respiratory failure sufficient to require non-invasive ventilation. We therefore used insertion of gastrostomy, or a vital capacity of 75% or less than predicted, as proxy markers of stage 4. The vital capacity threshold was selected on the basis of thresholds used in previous studies^13^, ^14^ and UK national guidelines.^15^ Because clinical stage was being estimated, and previous studies have not shown transition from later stages back to earlier stages, we maintained the highest stage recorded if a subsequent estimate of stage showed an apparent reversal. The lowest allocated clinical stage was stage 2 as the original trial consisted only of patients with El Escorial probable or definite ALS, and the analysis was therefore unable to answer questions about the effect of riluzole at stage 1 ALS. All enrolled patients provided informed consent for participation in the original clinical study and for use of the data collected for subsequent analyses. Ethics approval for the original trial was granted by the local or national independent ethics committee of each participating centre.

### Statistical analysis

Because stage at enrolment might differ between treatment groups and therefore affect analysis, we did a χ^2^ test of the independence of stage at enrolment and treatment group. To test the hypothesis that the benefit of riluzole treatment would be seen in all disease stages, we estimated the mean duration of each stage for each treatment group. We used the Kaplan-Meier product limit distribution to compare treatment groups for the time taken to change stage. The test was repeated, stratifying for stage at enrolment, and limited to participants entering at stage 2 or 3. We used Cox regression to confirm any finding of an effect of treatment group on time in clinical stage, controlling for covariates. Regression models were built stepwise, adding in stage at entry; an interaction term for treatment group and clinical stage at entry; age and sex, with covariates discarded if the model fit was not significantly improved.

We did several sensitivity analyses to ensure that the findings were robust. Kaplan-Meier analyses were repeated after combining riluzole treatment groups, either using the doses shown to significantly improve survival in the original efficacy trial (100 mg/day and 200 mg/day), using current treatment recommendations (100 mg per day), or using all tested doses (50 mg/day, 100 mg/day, and 200 mg/day), and with alternative vital capacity thresholds to define stage 4 of 70% or less and 80% or less of predicted. Since King's staging has not previously been used to estimate the timing of benefit, we also did the same analyses for the LiCALS data to exclude an artifact in trial data as a basis for the findings. To confirm the results were not an artifact of the analysis method, we also used a second approach, Multistate Outcome Analysis of Treatment (MOAT).^16^ MOAT does not tolerate missing data, and we therefore imputed missing or superseded disease stages by using the mean stage duration proportion by treatment groups across the study. Statistical tests were done using IBM SPSS Statistics 24.0, RStudio 1.0.143, R Foundation for Statistical Computing 3.4.1, and SAS 9.4. Original data from the riluzole trial can be accessed by application to Sanofi.

### Role of the funding source

The funders of the study had no role in study design and data collection, data analysis, data interpretation, or writing of the report. The corresponding author had full access to all the data in the study and had final responsibility for the decision to submit for publication.

## Results

**Figure 1:**
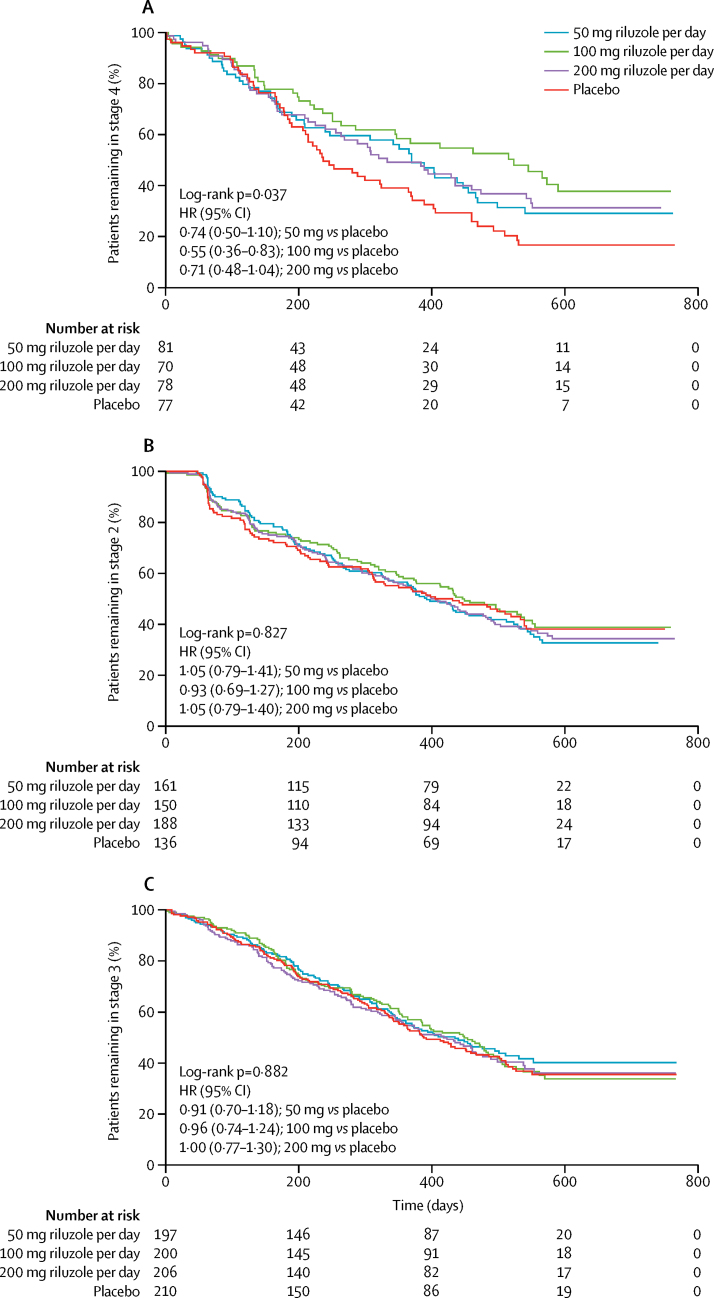
Patients progressing from each stage of amyotrophic lateral sclerosis with riluzole or placebo (A) Treatment with 100 mg riluzole per day significantly prolonged time in stage 4 compared with placebo (p=0·037). (B) Treatment with all doses did not prolong time in stage 2 compared with placebo (p=0·827). (C) Treatment with all doses did not prolong time in stage 3 compared with placebo (p=0·882). HR=hazard ratio.

We obtained data for all 959 patients assigned to treatment with case records from the original dose-ranging study; 237 assigned to riluzole 50 mg/day, 236 to 100 mg/day, 244 to 200 mg/day, and 242 to daily placebo. Three people were recorded as not taking trial medication, one assigned to 50 mg/day riluzole and two to 100 mg/day. 355 patients were enrolled at ALS stage 2, 451 at stage 3, and 153 at stage 4. Stage at enrolment did not differ between treatment groups (p=0·22; appendix). Counting the same patient at multiple stages where necessary, 355 patients reached stage 2, 678 reached stage 3, and 306 reached stage 4. Although there were no differences between treatment groups in the mean time spent transitioning to a later stage (table 1), time spent in stage 4 was longer for patients not transitioning who were receiving 100 mg/day riluzole than for those not transitioning who were receiving placebo (log-rank p=0·037; figure 1). Stratification by stage at enrolment using Kaplan-Meier analysis did not change this result (p=0·027). Results remained largely unchanged when the analysis was limited to those enrolling at stages 2 or 3 (appendix). Time from stages 2 or 3 to subsequent stages or death did not differ significantly between treatment groups and placebo (figure 1).

**Table 1:** Stage transition times for amyotrophic lateral sclerosis with riluzole or placebo

	**Stage 2**	**Stage 3**	**Stage 4**
**Time transitioning to a later stage or death**			
50 mg/day riluzole	256 (152)	241 (138)	224 (158)
100 mg/day riluzole	243 (161)	265 (150)	248 (180)
200 mg/day riluzole	243 (156)	230 (143)	233 (154)
Placebo	223 (157)	248 (143)	233 (154)
**Time maintaining the same stage over the trial**			
50 mg/day riluzole	570 (92)	492 (158)	404 (256)
100 mg/day riluzole	560 (88)	491 (160)	490 (230)
200 mg/day riluzole	576 (103)	450 (169)	507 (240)
Placebo	568 (90)	485 (170)	391 (288)

Data are mean (SD) times in days. Mean time spent was calculated per patient and averaged over all patients in that group.

Restricting the Kaplan-Meier analysis to the recommended treatment dose for riluzole of 100 mg/day still showed an extension of stage 4 (hazard ratio [HR] 0·53, 95% CI 0·35–0·81; p=0·003), which persisted after stratifying by stage at enrolment (p=0·003). Cox regression confirmed an effect of treatment group on time in stage 4 (p=0·009; table 2), independent of the effect of clinical stage at entry (p<0·0001) with no evidence for an effect of the interaction of treatment group and stage at entry, age, or sex. The findings were the same if treatment groups were tested in combination (treatment group p=0·006, stage at entry p<0·0001), or restricted to the recommended treatment dose of 100 mg per day (treatment group p<0·0001, stage at entry p<0·0001; table 2).

**Table 2:** Effect of variables on time spent in stage 4 amyotrophic lateral sclerosis

	**Hazard ratio**	**95% CI**	**p value**
**Individual treatment groups**			
Treatment effect overall	..	..	0·009
50 mg/day riluzole compared with placebo	0·742	0·501–1·100	0·138
100 mg/day riluzole compared with placebo	0·480	0·313–0·735	0·001
200 mg/day riluzole compared with placebo	0·710	0·481–1·048	0·085
Stage at entry effect overall	..	..	<0·0001
Entry at stage 2 compared with 4	1·697	1·063–2·709	0·027
Entry at stage 3 compared with 4	2·966	2·118–4·152	<0·0001
**Combined treatment groups compared with placebo**			
Treatment at any dose compared with placebo	0·638	0·464–0·878	0·006
Stage at entry effect overall	..	..	<0·0001
Entry at stage 2 compared with 4	1·664	1·043–2·655	0·033
Entry at stage 3 compared with 4	2·825	2·026–3·939	<0·0001
**Recommended treatment dose compared with placebo**			
100 mg/day riluzole compared with placebo	0·456	0·295–0·704	0·0004
Stage at entry effect overall	..	..	<0·0001
Entry at stage 2 compared with 4	2·034	1·031–4·016	0·041
Entry at stage 3 compared with 4	3·154	1·957–5·080	<0·0001
**Combined higher treatment doses compared with placebo**			
100 mg/day or 200 mg/day riluzole compared with placebo	0·578	0·409–0·816	0·002
Stage at entry effect overall	..	..	<0·0001
Entry at stage 2 compared with 4	2·268	1·357–3·790	0·002
Entry at stage 3 compared with 4	3·023	2·055–4·447	<0·0001

Data analysed by Cox regression. Variables were included step-wise in the model and removed if there was no significant improvement in the model fit. The variables tested were treatment group; stage at trial entry; interaction between treatment group and stage at trial entry; age; and sex. Only treatment group and stage at trial entry were retained in the model.

**Figure 2:**
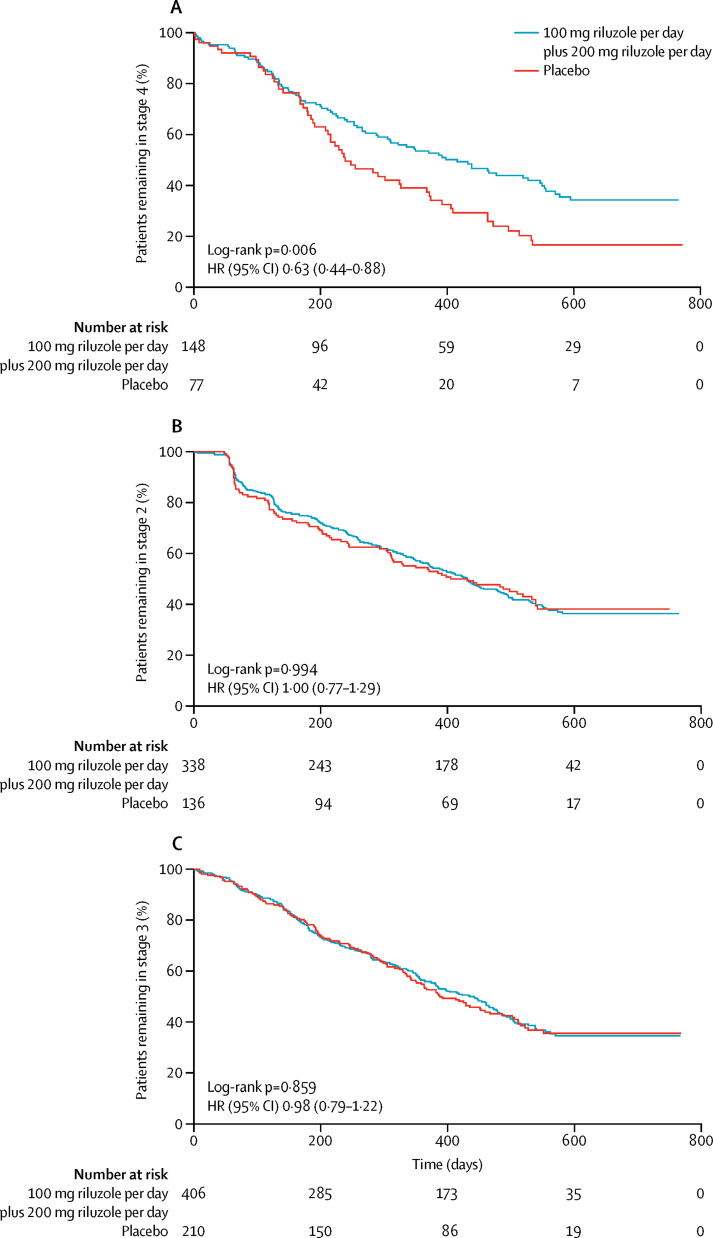
Patients progressing from each stage of amyotrophic lateral sclerosis with 100 mg plus 200 mg riluzole or placebo (A) Treatment with higher doses significantly prolonged time in stage 4 compared with placebo (p=0·006). (B) Treatment with higher doses did not prolong time in stage 2 compared with placebo (p=0·994). (C) Treatment with higher doses did not prolong time in stage 3 compared with placebo (p=0·859). HR=hazard ratio.

Combining treatment groups did not change these results. Analysing all treatment groups as a whole against placebo showed a significant prolongation of stage 4 in the treatment groups (HR 0·66, 95% CI 0·48–0·91; p=0·01), as did limiting the analysis to the two higher doses against placebo (figure 2), but there was no prolongation for other stages (figure 2). Altering the vital capacity threshold defining stage 4 to 80% or less did not change the findings (p=0·014), although reducing it to 70% or less of predicted meant that only 39 patients fulfilled respiratory criteria for stage 4 and the effect of prolonging stage 4 with treatment was no longer evident (HR 50 mg *vs* placebo 0·84 [0·51–1·37], 100 mg *vs* placebo 0·60 [0·36–1·00], 200 mg *vs* placebo 0·67 [0·42–1·08]; p=0·18). The findings were unchanged when treatment groups were combined, regardless of the definition of stage 4: for vital capacity of 70% or less than predicted, higher doses versus placebo HR 0·64 (95% CI 0·42–0·98), p=0·037, all doses versus placebo 0·69 (0·47–1·03), p=0·067; for vital capacity of 80% or less than predicted, higher doses versus placebo 0·66 (0·51–0·86), p=0·002, all doses versus placebo 0·68 (0·54–0·87), p=0·002.

To exclude an artifactual explanation for findings, we did comparison tests using data from the LiCALS trial in which all 217 participants entered at stage 1, of which 214 were randomly assigned, 107 to treatment and 107 to placebo. Treatment with lithium did not prolong the duration of any stage (for stage 1, HR 1·00, 95% CI 0·84–1·19, p=0·98; stage 2, 1·04, 0·83–1·30, p=0·73, stage 3, 1·40, 0·96–2·04, p=0·082, and stage 4, 1·51, 0·74–3·05, p=0·25). MOAT analysis confirmed the findings of the Kaplan-Meier approach, showing that those treated with riluzole had a longer stage 4 than those on placebo (table 3).

**Table 3:** Multistate outcome analysis of treatment analysis of time to transition from one stage of amyotrophic lateral sclerosis to the next

		**50 mg/day riluzole**	**100 mg/day riluzole**	**200 mg/day riluzole**	**Placebo**
Stage transition					
	2–3	109 (99–118)	70 (60–81)	100 (89–110)	82 (72–91)
	3–4	38 (29–48)	52 (43–61)	30 (23–37)	69 (61–78)
	4–5	207 (195–219)	234 (222–246)	226 (215–237)	198 (186–209)

Data are the mean number of days (95% CI), presented by treatment group.

## Discussion

Treatment with riluzole prolonged stage 4 in patients with ALS. This result was robust to the method of analysis and independent of the stage at which treatment was started. This finding implies that the survival benefit of riluzole is achieved by extending stage 4, not by prolonging stages 2 or 3, or generally slowing disease.

Patients counselled about riluzole are told that it extends life, but not at which stage, since this was not clear from the original study.^10^ One analysis suggested that benefit might occur while function is well preserved,^17^ but the dose-ranging riluzole trial showed no overall effect on function,^10^ which led to the conundrum of how to explain an improvement in survival without a concomitant effect on function. Our finding that the extension of life is due to an extension of stage 4 helps to resolve this confusion, since function at this stage is limited, and a flattening of the slope of functional decline would be hard to detect. Furthermore, the ALSFRS-R slope change with time is curvilinear and therefore flattens at the beginning and end.^18^ Although the timing might seem undesirable since the prolongation of life is when disability is high, rather than when the patient is functionally well, all other current treatments that extend life act at the last stage of disease. Non-invasive ventilation for example, has been shown to prolong life and improve quality,^19^ and is used at stage 4. The take-up of non-invasive ventilation is high among patients,^20^ suggesting that prolongation of life at later stages of disease is not undesirable in itself, and riluzole is well tolerated in advanced ALS.^21^ Similarly, gastrostomy is used to support those with nutritional failure due to dysphagia, improving quality of life,^22^ and also is applied at stage 4.

A direct clinical implication of our findings is that patients can be told that riluzole extends the later stages of ALS, but it is important to note that it might also extend stage 1, since we have no information on this stage from the trial data.

Riluzole could act through several mechanisms; eg, on excitotoxic pathways early in the course of ALS, and through effects on mitochondrial function, fat metabolism, or diaphragmatic strength that might be more crucial to survival later. A specific benefit of riluzole therapy in patients with reduced vital capacity is supported by statistical model-based analysis of clinical trial data.^23^ When riluzole was first identified as a beneficial treatment for ALS, its use in various health-care systems was controversial because the survival benefit was seen as small, while the drug cost was seen as high. A combination of health-economics analyses and pressure from patient groups led to its widespread adoption,^24^ although in some countries approval was delayed. In the UK, the National Institute for Health and Care Excellence approved riluzole for ALS following a detailed cost-benefit analysis that included the concept of quality-adjusted life-years.^25^, ^26^ Although our finding of a prolongation of stage 4 might affect such analyses, riluzole is no longer on patent; it is now cheaper than it was when the initial health-economics analyses were done, and cheaper than other treatments for ALS, such as edaravone.^27^

A strength of our study was the use of clinical staging to analyse clinical trial data in a neurodegenerative disease, allowing an examination of when benefit occurs in a way that is easily understood by clinicians and patients. Thus, as an outcome measure, clinical staging has an important part to play in future trial design in ALS and other neurodegenerative diseases. In cancers, another group of diseases which if untreated lead to progressive disability and death, trials routinely use staging to decide on the appropriate treatment and to assess outcome.^28^ A further benefit of staging is that successful treatments can be shown to reverse the progression through clinical stages.

There were several important weaknesses of this study. It was a post-hoc analysis, and therefore did not provide the same level of evidence as a prespecified analysis, since the study design did not consider staging in the calculation of statistical power or in the assessment criteria. Furthermore, the criteria for stage 4 mean that 153 (16%) of the 959 patients were in stage 4 at enrolment. This would not usually be the case in a modern trial in ALS given the stringent criteria commonly used now (eg, forced vital capacity of ≥80%). However, our findings were similar when these 153 patients were excluded in a further analysis. Clinical stage was estimated from trial data. We have previous experience in this process, and have successfully applied an algorithm to the ALSFRS-R to derive clinical stage.^6^ In this study, we could not use the ALSFRS-R because such a scale did not exist when the trial data were collected; as a result, we had to generate a new algorithm to estimate clinical stage. There was no way to validate this new algorithm, since one of the scales it used, the Norris scale, is no longer in use. To overcome this, we applied the same logical process to the data that was used to generate the ALSFRS-R algorithm for staging. Furthermore, adjusting the criteria defining the clinical stages did not change the findings of the study, and using two entirely different analytical approaches generated the same conclusions.

A further limitation of this study arose from the strict inclusion criteria of the original trial, which was restricted to people fulfilling El Escorial criteria for probable or definite ALS.^10^ This prevented our study from analysing the treatment effects of riluzole in stage 1 of disease. However, some studies have suggested that the effects of riluzole might be transient,^29^, ^30^, ^31^ and support treatment in the early stages of ALS.^30^ To determine whether riluzole extends stage 1 will require a specific trial. Additionally, patients' weight was not recorded at each visit in the original study and therefore the nutritional component of the stage 4 definition was inferred from the date of gastrostomy. Our findings therefore need to be validated in a future trial with regular recording of weight. Although it is ethically difficult to do new studies exclusively of riluzole, studies of this drug embedded within other clinical trials have been completed (eg, the study of dexpramipexole in ALS; EMPOWER, NCT01281189),^32^ or are underway (eg, a study of low-dose interleukin-2 in ALS; MIROCALS, NCT03039673). Such an approach could potentially address our findings within a prospective study design, or retrospectively confirm these findings using similar techniques to ours within existing data.

Riluzole is currently the only treatment shown to prolong life in patients with ALS. We have shown that it acts by prolonging stage 4 ALS rather than by slowing the entire disease course or prolonging intermediate stages. Similar methods should be used in future clinical trials of ALS or other neurodegenerative diseases where survival is an endpoint, to show where benefit is accrued and to allow a full discussion of effects when counselling patients about treatment.
