# Supplementary material for: Stage at which riluzole treatment prolongs survival in patients with amyotrophic lateral sclerosis: a retrospective analysis of data from a dose-ranging study
Source: Lancet Neurol. 2018 May;17(5):416–22. doi: 10.1016/S1474-4422(18)30054-1 (PMC5899963; doi:10.1016/S1474-4422(18)30054-1)
Supplement: Supplementary appendix [file mmc1.pdf]

THE LANCET
Neurology

Supplementary appendix

This appendix formed part of the original submission and has been peer reviewed. We post it as supplied by the authors.

Supplement to: Fang T, Al Khleifat A, Meurgey J-H, et al. Stage at which riluzole treatment prolongs survival in patients with amyotrophic lateral sclerosis: a retrospective analysis of data from a dose-ranging study. *Lancet Neurol* 2018; published online March 7. http://dx.doi.org/10.1016/S1474-4422(18)30054-1.

Staging algorithm

A regional analysis was performed, using regions defined as Bulbar, Upper limb, Lower limb.

- Stages 1-3 were scored based on the number of affected regions. A region was considered affected if there was a drop in maximum score for that region in any of the relevant scales (Norris bulbar, Norris limb and muscle strength). One affected region = stage 1, two affected regions = stage 2, three affected regions = stage 3. The minimum stage at entry was stage 2 because the minimum El Escorial category at entry was Probable, which requires two regions to be affected.
- Stage 4 was scored if the person had a gastrostomy, defined by the date of insertion, or vital capacity ≤75% predicted value, whichever date fell earlier.
- Stage 5 was scored as the date of death, tracheostomy or intubation, whichever date fell earlier.

The censor date was 31 December 1994. Any events occurring after this date were not recorded and treated as censored.

No stage reversals were allowed.

Treatment	**Stage 2**	**Stage 3**	**Stage 4**
50mg/day	90	106	41
100mg/day	87	117	32
200mg/day	101	101	42
Placebo	77	127	38

Supplementary Table 1: **Stage at enrolment by treatment group.** There was no significant difference between treatment groups for the number of patients enrolling at each stage (P = 0.22).

Treatment combinations vs placebo	*P*-value
All doses	0.053
Higher doses combined	0.043
All doses combined	0.027

Supplementary Table 2: **Kaplan Meier product limit distribution of the effect of Riluzole on Stage 4 for participants with a stage of enrolment of 2 or 3.** All doses vs placebo (50mg, 100mg, 200mg individually vs placebo), higher doses combined vs placebo (100mg + 200mg vs placebo) and all doses combined vs placebo (50mg + 100mg + 200mg vs placebo).
